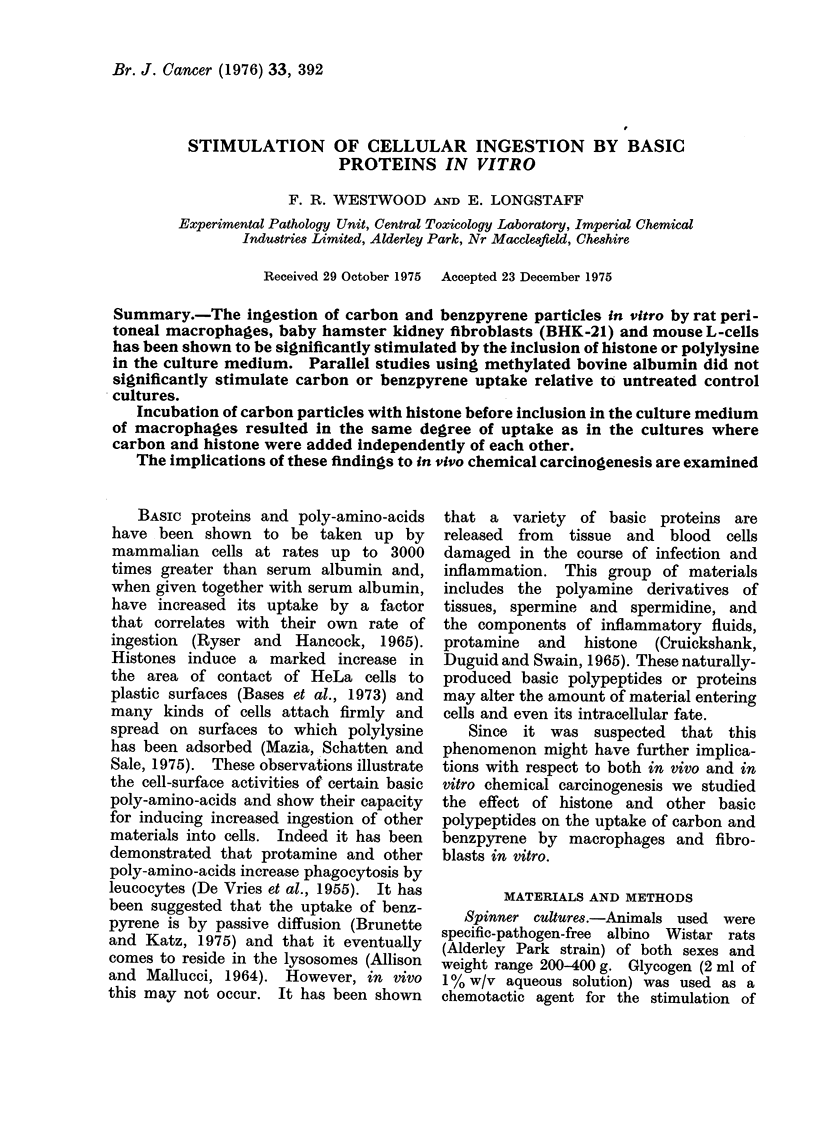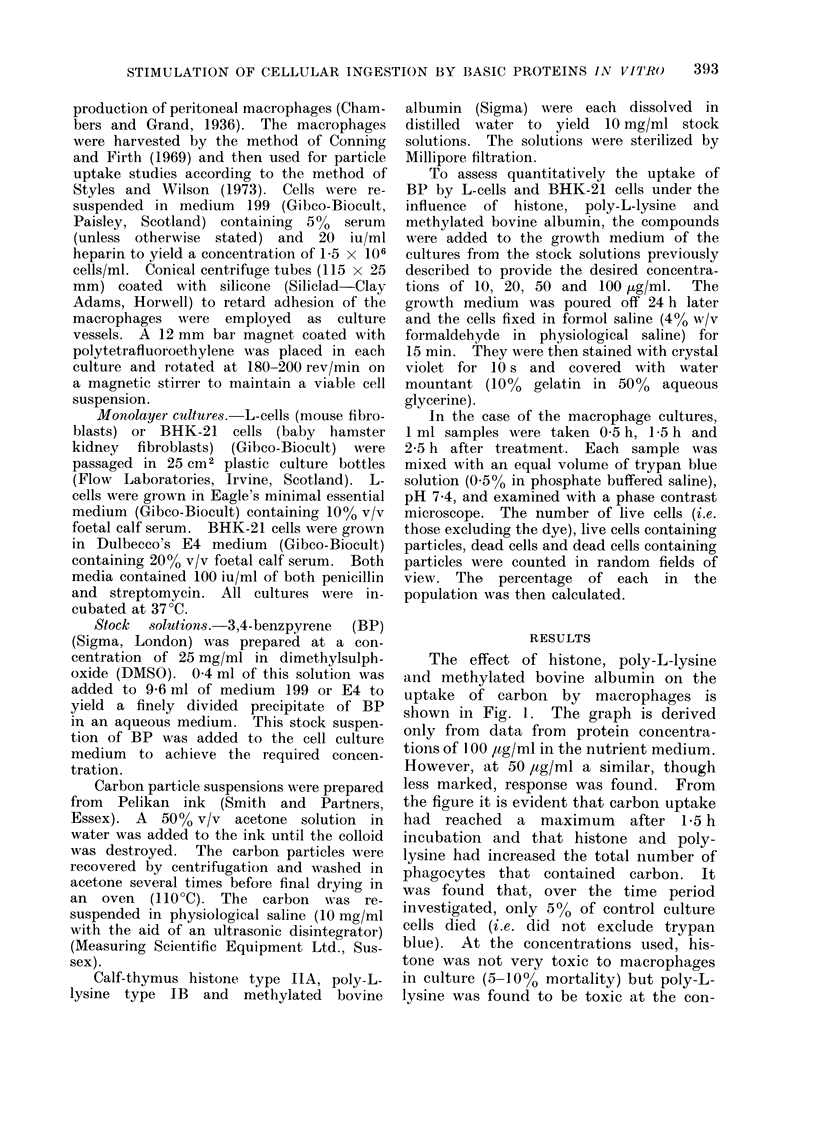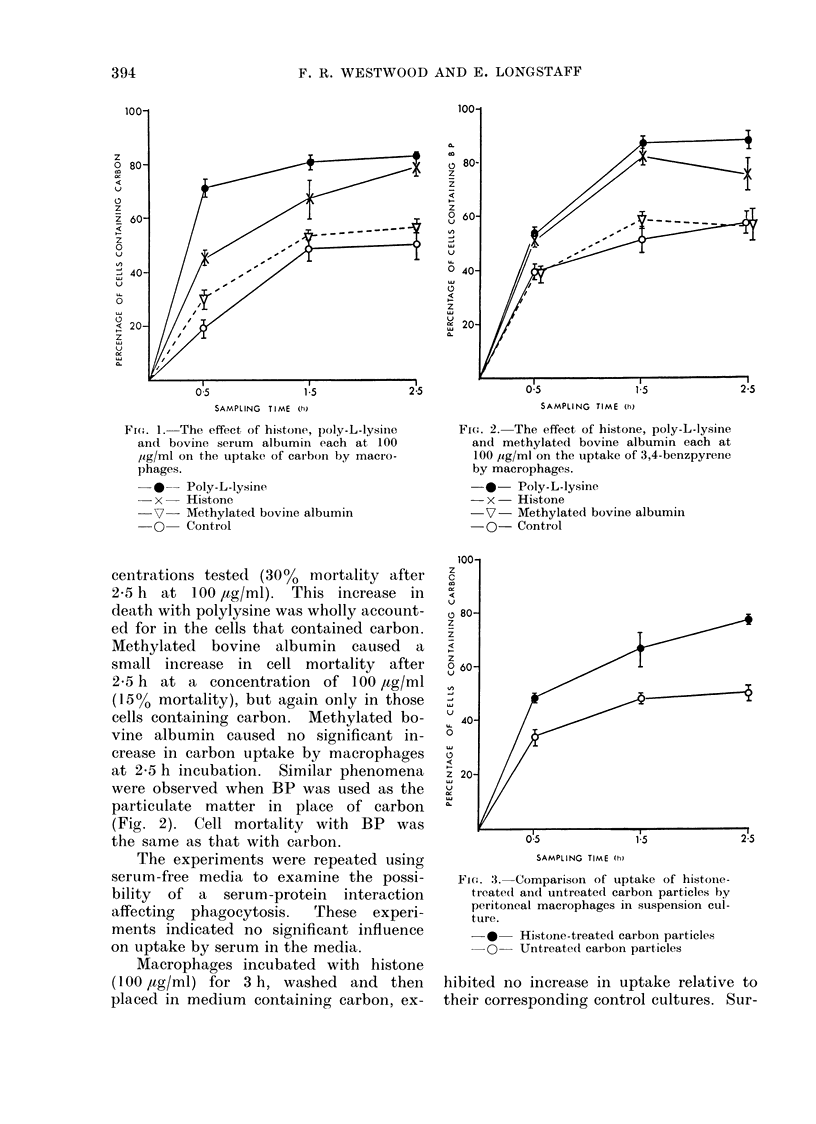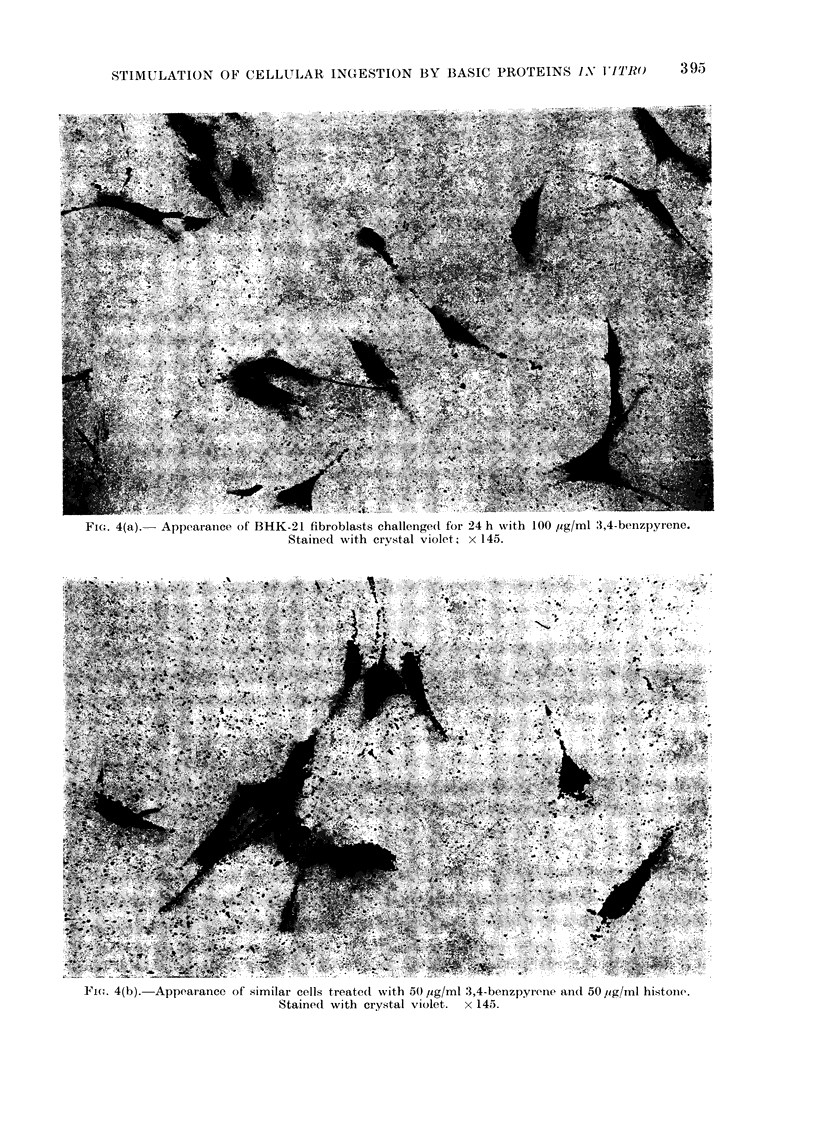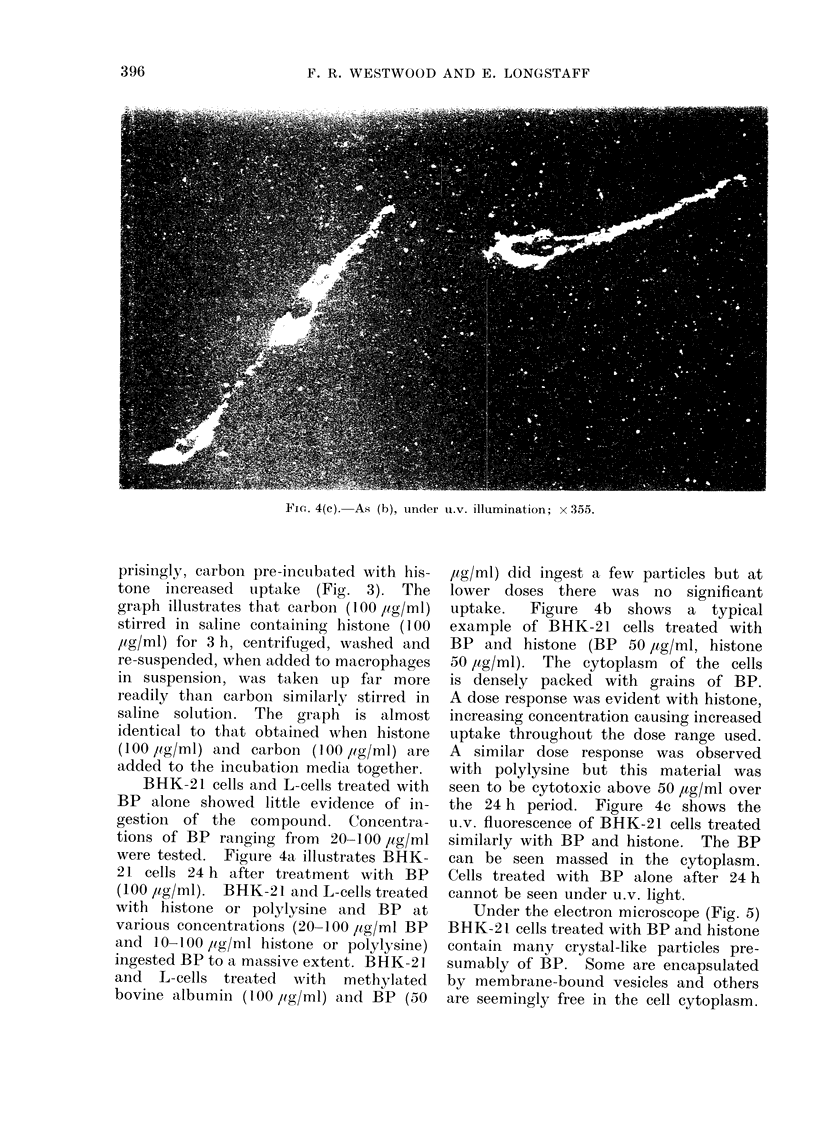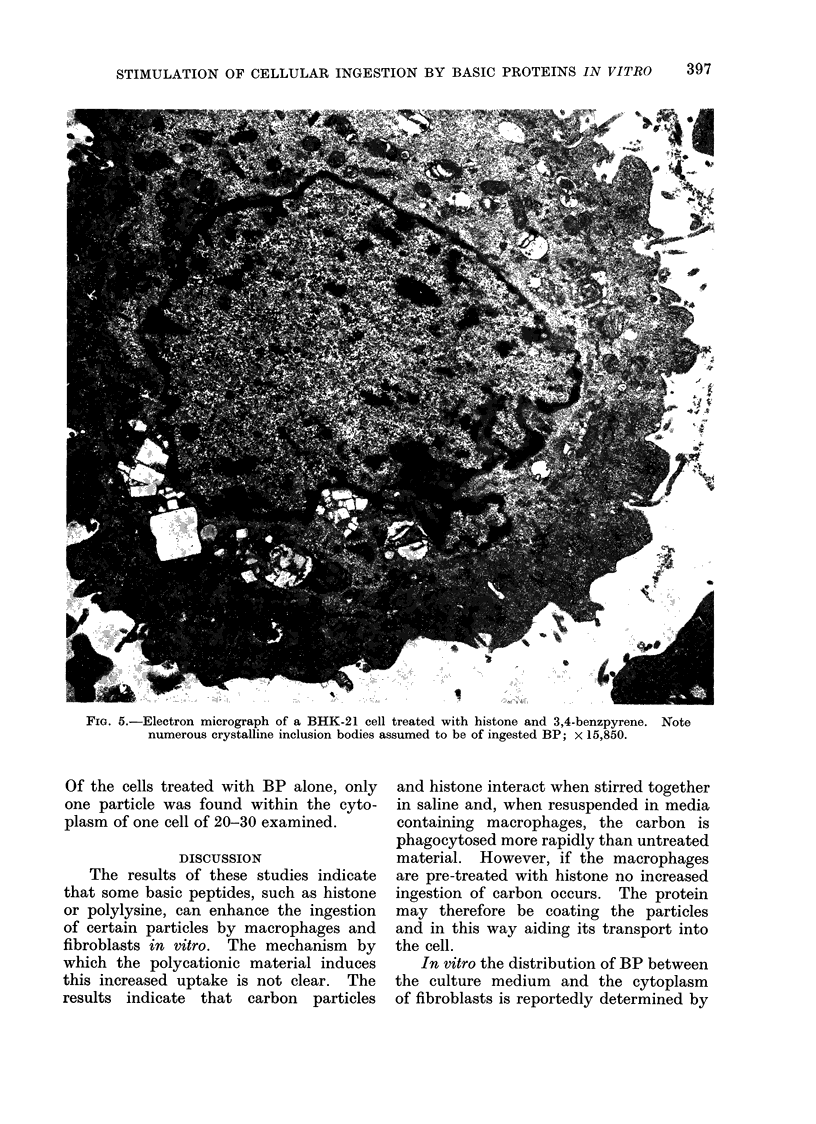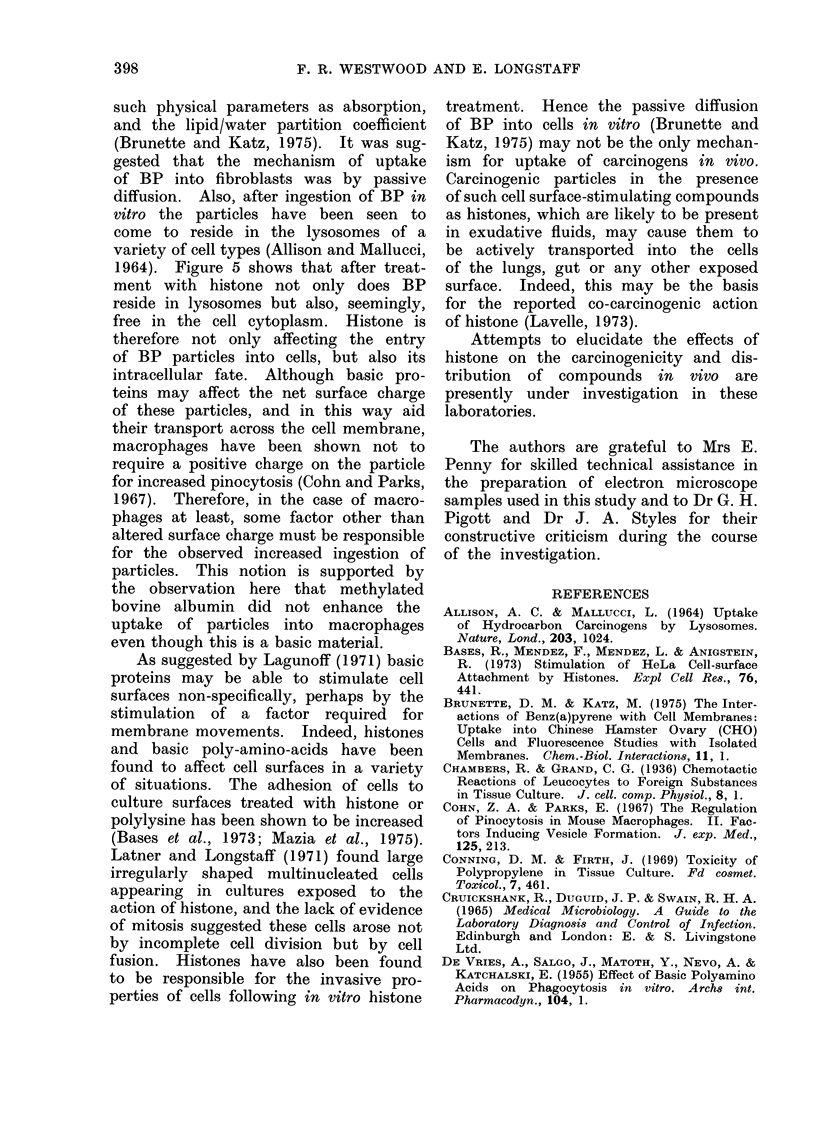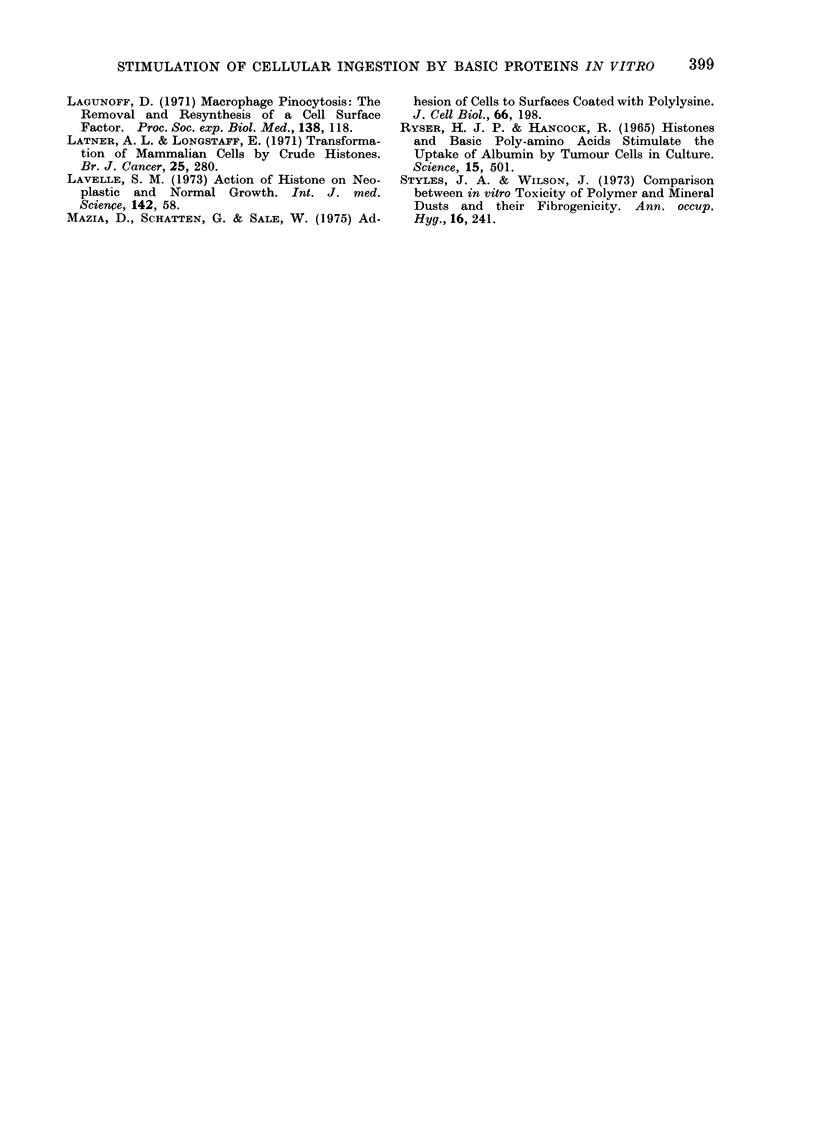# Stimulation of cellular ingestion by basic proteins in vitro.

**DOI:** 10.1038/bjc.1976.64

**Published:** 1976-04

**Authors:** F. R. Westwood, E. Longstaff

## Abstract

**Images:**


*Br. J. Cancer* (1976) **33**, 392

### STIMULATION OF CELLULAR INGESTION BY BASIC PROTEINS *IN VITRO*

F. R. WESTWOOD AND E. LONGSTAFF

*Experimental Pathology Unit, Central Toxicology Laboratory, Imperial Chemical Industries Limited, Alderley Park, Nr Macclesfield, Cheshire*

Received 29 October 1975 Accepted 23 December 1975

**Summary.—The ingestion of carbon and benzpyrene particles *in vitro* by rat peritoneal macrophages, baby hamster kidney fibroblasts (BHK-21) and mouse L-cells has been shown to be significantly stimulated by the inclusion of histone or polylysine in the culture medium. Parallel studies using methylated bovine albumin did not significantly stimulate carbon or benzpyrene uptake relative to untreated control cultures.**

**Incubation of carbon particles with histone before inclusion in the culture medium of macrophages resulted in the same degree of uptake as in the cultures where carbon and histone were added independently of each other.**

**The implications of these findings to *in vivo* chemical carcinogenesis are examined**

Basic proteins and poly-amino-acids have been shown to be taken up by mammalian cells at rates up to 3000 times greater than serum albumin and, when given together with serum albumin, have increased its uptake by a factor that correlates with their own rate of ingestion (Ryser and Hancock, 1965). Histones induce a marked increase in the area of contact of HeLa cells to plastic surfaces (Bases *et al.*, 1973) and many kinds of cells attach firmly and spread on surfaces to which polylysine has been adsorbed (Mazia, Schatten and Sale, 1975). These observations illustrate the cell-surface activities of certain basic poly-amino-acids and show their capacity for inducing increased ingestion of other materials into cells. Indeed it has been demonstrated that protamine and other poly-amino-acids increase phagocytosis by leucocytes (De Vries *et al.*, 1955). It has been suggested that the uptake of benzpyrene is by passive diffusion (Brunette and Katz, 1975) and that it eventually comes to reside in the lysosomes (Allison and Mallucci, 1964). However, *in vivo* this may not occur. It has been shown that a variety of basic proteins are released from tissue and blood cells damaged in the course of infection and inflammation. This group of materials includes the polyamine derivatives of tissues, spermine and spermidine, and the components of inflammatory fluids, protamine and histone (Cruickshank, Duguid and Swain, 1965). These naturally-produced basic polypeptides or proteins may alter the amount of material entering cells and even its intracellular fate.

Since it was suspected that this phenomenon might have further implications with respect to both *in vivo* and *in vitro* chemical carcinogenesis we studied the effect of histone and other basic polypeptides on the uptake of carbon and benzpyrene by macrophages and fibroblasts *in vitro*.

### MATERIALS AND METHODS

*Spinner cultures.*—Animals used were specific-pathogen-free albino Wistar rats (Alderley Park strain) of both sexes and weight range 200–400 g. Glycogen (2 ml of 1% w/v aqueous solution) was used as a chemotactic agent for the stimulation of production of peritoneal macrophages (Chambers and Grand, 1936). The macrophages were harvested by the method of Conning and Firth (1969) and then used for particle uptake studies according to the method of Styles and Wilson (1973). Cells were resuspended in medium 199 (Gibco-Biocult, Paisley, Scotland) containing 5% serum (unless otherwise stated) and 20 iu/ml heparin to yield a concentration of $1{\cdot}5 \times 10^6$ cells/ml. Conical centrifuge tubes ($115 \times 25$ mm) coated with silicone (Siliclad—Clay Adams, Horwell) to retard adhesion of the macrophages were employed as culture vessels. A 12 mm bar magnet coated with polytetrafluoroethylene was placed in each culture and rotated at 180–200 rev/min on a magnetic stirrer to maintain a viable cell suspension.

*Monolayer cultures.*—L-cells (mouse fibroblasts) or BHK-21 cells (baby hamster kidney fibroblasts) (Gibco-Biocult) were passaged in 25 $cm^2$ plastic culture bottles (Flow Laboratories, Irvine, Scotland). L-cells were grown in Eagle's minimal essential medium (Gibco-Biocult) containing 10% v/v foetal calf serum. BHK-21 cells were grown in Dulbecco's E4 medium (Gibco-Biocult) containing 20% v/v foetal calf serum. Both media contained 100 iu/ml of both penicillin and streptomycin. All cultures were incubated at 37 °C.

*Stock solutions.*—3,4-benzpyrene (BP) (Sigma, London) was prepared at a concentration of 25 mg/ml in dimethylsulphoxide (DMSO). 0·4 ml of this solution was added to 9·6 ml of medium 199 or E4 to yield a finely divided precipitate of BP in an aqueous medium. This stock suspension of BP was added to the cell culture medium to achieve the required concentration.

Carbon particle suspensions were prepared from Pelikan ink (Smith and Partners, Essex). A 50% v/v acetone solution in water was added to the ink until the colloid was destroyed. The carbon particles were recovered by centrifugation and washed in acetone several times before final drying in an oven (110°C). The carbon was resuspended in physiological saline (10 mg/ml with the aid of an ultrasonic disintegrator) (Measuring Scientific Equipment Ltd., Sussex).

Calf-thymus histone type IIA, poly-L-lysine type IB and methylated bovine albumin (Sigma) were each dissolved in distilled water to yield 10 mg/ml stock solutions. The solutions were sterilized by Millipore filtration.

To assess quantitatively the uptake of BP by L-cells and BHK-21 cells under the influence of histone, poly-L-lysine and methylated bovine albumin, the compounds were added to the growth medium of the cultures from the stock solutions previously described to provide the desired concentrations of 10, 20, 50 and 100 $\mu$g/ml. The growth medium was poured off 24 h later and the cells fixed in formol saline (4% w/v formaldehyde in physiological saline) for 15 min. They were then stained with crystal violet for 10 s and covered with water mountant (10% gelatin in 50% aqueous glycerine).

In the case of the macrophage cultures, 1 ml samples were taken 0·5 h, 1·5 h and 2·5 h after treatment. Each sample was mixed with an equal volume of trypan blue solution (0·5% in phosphate buffered saline), pH 7·4, and examined with a phase contrast microscope. The number of live cells (*i.e.* those excluding the dye), live cells containing particles, dead cells and dead cells containing particles were counted in random fields of view. The percentage of each in the population was then calculated.

### RESULTS

The effect of histone, poly-L-lysine and methylated bovine albumin on the uptake of carbon by macrophages is shown in Fig. 1. The graph is derived only from data from protein concentrations of 100 $\mu$g/ml in the nutrient medium. However, at 50 $\mu$g/ml a similar, though less marked, response was found. From the figure it is evident that carbon uptake had reached a maximum after 1·5 h incubation and that histone and polylysine had increased the total number of phagocytes that contained carbon. It was found that, over the time period investigated, only 5% of control culture cells died (*i.e.* did not exclude trypan blue). At the concentrations used, histone was not very toxic to macrophages in culture (5–10% mortality) but poly-L-lysine was found to be toxic at the con-

FIG. 1.—The effect of histone, poly-L-lysine and bovine serum albumin each at 100 $\mu$g/ml on the uptake of carbon by macrophages.

—●— Poly-L-lysine

—×— Histone

—▽— Methylated bovine albumin

—○— Control

FIG. 2.—The effect of histone, poly-L-lysine and methylated bovine albumin each at 100 $\mu$g/ml on the uptake of 3,4-benzpyrene by macrophages.

—●— Poly-L-lysine

—×— Histone

—▽— Methylated bovine albumin

—○— Control centrations tested (30% mortality after 2·5 h at 100 $\mu$g/ml). This increase in death with polylysine was wholly accounted for in the cells that contained carbon. Methylated bovine albumin caused a small increase in cell mortality after 2·5 h at a concentration of 100 $\mu$g/ml (15% mortality), but again only in those cells containing carbon. Methylated bovine albumin caused no significant increase in carbon uptake by macrophages at 2·5 h incubation. Similar phenomena were observed when BP was used as the particulate matter in place of carbon (Fig. 2). Cell mortality with BP was the same as that with carbon.

The experiments were repeated using serum-free media to examine the possibility of a serum-protein interaction affecting phagocytosis. These experiments indicated no significant influence on uptake by serum in the media.

FIG. 3.—Comparison of uptake of histone-treated and untreated carbon particles by peritoneal macrophages in suspension culture.

—●— Histone-treated carbon particles
—○— Untreated carbon particles

Macrophages incubated with histone (100 $\mu$g/ml) for 3 h, washed and then placed in medium containing carbon, exhibited no increase in uptake relative to their corresponding control cultures. Sur-

FIG. 4(a).— Appearance of BHK-21 fibroblasts challenged for 24 h with 100 $\mu$g/ml 3,4-benzpyrene. Stained with crystal violet; $\times$ 145.

FIG. 4(b).—Appearance of similar cells treated with 50 $\mu$g/ml 3,4-benzpyrene and 50 $\mu$g/ml histone. Stained with crystal violet. $\times$ 145.

FIG. 4(c).—As (b), under u.v. illumination; ×355.

prisingly, carbon pre-incubated with histone increased uptake (Fig. 3). The graph illustrates that carbon (100 μg/ml) stirred in saline containing histone (100 μg/ml) for 3 h, centrifuged, washed and re-suspended, when added to macrophages in suspension, was taken up far more readily than carbon similarly stirred in saline solution. The graph is almost identical to that obtained when histone (100 μg/ml) and carbon (100 μg/ml) are added to the incubation media together.

BHK-21 cells and L-cells treated with BP alone showed little evidence of ingestion of the compound. Concentrations of BP ranging from 20–100 μg/ml were tested. Figure 4a illustrates BHK-21 cells 24 h after treatment with BP (100 μg/ml). BHK-21 and L-cells treated with histone or polylysine and BP at various concentrations (20–100 μg/ml BP and 10–100 μg/ml histone or polylysine) ingested BP to a massive extent. BHK-21 and L-cells treated with methylated bovine albumin (100 μg/ml) and BP (50 μg/ml) did ingest a few particles but at lower doses there was no significant uptake. Figure 4b shows a typical example of BHK-21 cells treated with BP and histone (BP 50 μg/ml, histone 50 μg/ml). The cytoplasm of the cells is densely packed with grains of BP. A dose response was evident with histone, increasing concentration causing increased uptake throughout the dose range used. A similar dose response was observed with polylysine but this material was seen to be cytotoxic above 50 μg/ml over the 24 h period. Figure 4c shows the u.v. fluorescence of BHK-21 cells treated similarly with BP and histone. The BP can be seen massed in the cytoplasm. Cells treated with BP alone after 24 h cannot be seen under u.v. light.

Under the electron microscope (Fig. 5) BHK-21 cells treated with BP and histone contain many crystal-like particles presumably of BP. Some are encapsulated by membrane-bound vesicles and others are seemingly free in the cell cytoplasm.

FIG. 5.—Electron micrograph of a BHK-21 cell treated with histone and 3,4-benzpyrene. Note numerous crystalline inclusion bodies assumed to be of ingested BP; × 15,850.

Of the cells treated with BP alone, only one particle was found within the cytoplasm of one cell of 20–30 examined.

### DISCUSSION

The results of these studies indicate that some basic peptides, such as histone or polylysine, can enhance the ingestion of certain particles by macrophages and fibroblasts *in vitro*. The mechanism by which the polycationic material induces this increased uptake is not clear. The results indicate that carbon particles and histone interact when stirred together in saline and, when resuspended in media containing macrophages, the carbon is phagocytosed more rapidly than untreated material. However, if the macrophages are pre-treated with histone no increased ingestion of carbon occurs. The protein may therefore be coating the particles and in this way aiding its transport into the cell.

*In vitro* the distribution of BP between the culture medium and the cytoplasm of fibroblasts is reportedly determined by such physical parameters as absorption, and the lipid/water partition coefficient (Brunette and Katz, 1975). It was suggested that the mechanism of uptake of BP into fibroblasts was by passive diffusion. Also, after ingestion of BP *in vitro* the particles have been seen to come to reside in the lysosomes of a variety of cell types (Allison and Mallucci, 1964). Figure 5 shows that after treatment with histone not only does BP reside in lysosomes but also, seemingly, free in the cell cytoplasm. Histone is therefore not only affecting the entry of BP particles into cells, but also its intracellular fate. Although basic proteins may affect the net surface charge of these particles, and in this way aid their transport across the cell membrane, macrophages have been shown not to require a positive charge on the particle for increased pinocytosis (Cohn and Parks, 1967). Therefore, in the case of macrophages at least, some factor other than altered surface charge must be responsible for the observed increased ingestion of particles. This notion is supported by the observation here that methylated bovine albumin did not enhance the uptake of particles into macrophages even though this is a basic material.

As suggested by Lagunoff (1971) basic proteins may be able to stimulate cell surfaces non-specifically, perhaps by the stimulation of a factor required for membrane movements. Indeed, histones and basic poly-amino-acids have been found to affect cell surfaces in a variety of situations. The adhesion of cells to culture surfaces treated with histone or polylysine has been shown to be increased (Bases *et al.*, 1973; Mazia *et al.*, 1975). Latner and Longstaff (1971) found large irregularly shaped multinucleated cells appearing in cultures exposed to the action of histone, and the lack of evidence of mitosis suggested these cells arose not by incomplete cell division but by cell fusion. Histones have also been found to be responsible for the invasive properties of cells following *in vitro* histone treatment. Hence the passive diffusion of BP into cells *in vitro* (Brunette and Katz, 1975) may not be the only mechanism for uptake of carcinogens *in vivo*. Carcinogenic particles in the presence of such cell surface-stimulating compounds as histones, which are likely to be present in exudative fluids, may cause them to be actively transported into the cells of the lungs, gut or any other exposed surface. Indeed, this may be the basis for the reported co-carcinogenic action of histone (Lavelle, 1973).

Attempts to elucidate the effects of histone on the carcinogenicity and distribution of compounds *in vivo* are presently under investigation in these laboratories.

The authors are grateful to Mrs E. Penny for skilled technical assistance in the preparation of electron microscope samples used in this study and to Dr G. H. Pigott and Dr J. A. Styles for their constructive criticism during the course of the investigation.

### REFERENCES

Allison, A. C. & Mallucci, L. (1964) Uptake of Hydrocarbon Carcinogens by Lysosomes. *Nature, Lond.*, **203**, 1024.

Bases, R., Mendez, F., Mendez, L. & Anigstein, R. (1973) Stimulation of HeLa Cell-surface Attachment by Histones. *Expl Cell Res.*, **76**, 441.

Brunette, D. M. & Katz, M. (1975) The Interactions of Benz(a)pyrene with Cell Membranes: Uptake into Chinese Hamster Ovary (CHO) Cells and Fluorescence Studies with Isolated Membranes. *Chem.-Biol. Interactions*, **11**, 1.

Chambers, R. & Grand, C. G. (1936) Chemotactic Reactions of Leucocytes to Foreign Substances in Tissue Culture. *J. cell. comp. Physiol.*, **8**, 1.

Cohn, Z. A. & Parks, E. (1967) The Regulation of Pinocytosis in Mouse Macrophages. II. Factors Inducing Vesicle Formation. *J. exp. Med.*, **125**, 213.

Conning, D. M. & Firth, J. (1969) Toxicity of Polypropylene in Tissue Culture. *Fd cosmet. Toxicol.*, **7**, 461.

Cruickshank, R., Duguid, J. P. & Swain, R. H. A. (1965) *Medical Microbiology. A Guide to the Laboratory Diagnosis and Control of Infection.* Edinburgh and London: E. & S. Livingstone Ltd.

De Vries, A., Salgo, J., Matoth, Y., Nevo, A. & Katchalski, E. (1955) Effect of Basic Polyamino Acids on Phagocytosis *in vitro*. *Archs int. Pharmacodyn.*, **104**, 1.

LAGUNOFF, D. (1971) Macrophage Pinocytosis: The Removal and Resynthesis of a Cell Surface Factor. *Proc. Soc. exp. Biol. Med.*, **138**, 118.

LATNER, A. L. & LONGSTAFF, E. (1971) Transformation of Mammalian Cells by Crude Histones. *Br. J. Cancer*, **25**, 280.

LAVELLE, S. M. (1973) Action of Histone on Neoplastic and Normal Growth. *Int. J. med. Science*, **142**, 58.

MAZIA, D., SCHATTEN, G. & SALE, W. (1975) Adhesion of Cells to Surfaces Coated with Polylysine. *J. Cell Biol.*, **66**, 198.

RYSER, H. J. P. & HANCOCK, R. (1965) Histones and Basic Poly-amino Acids Stimulate the Uptake of Albumin by Tumour Cells in Culture. *Science*, **15**, 501.

STYLES, J. A. & WILSON, J. (1973) Comparison between *in vitro* Toxicity of Polymer and Mineral Dusts and their Fibrogenicity. *Ann. occup. Hyg.*, **16**, 241.